# Comparing the Use of Spatially Explicit Indicators and Conventional Indicators in the Evaluation of Healthy Cities: A Case Study in Shenzhen, China

**DOI:** 10.3390/ijerph17207409

**Published:** 2020-10-12

**Authors:** Jun Yang, Xiangyu Luo, Yixiong Xiao, Shaoqing Shen, Mo Su, Yuqi Bai, Peng Gong

**Affiliations:** 1 Key Laboratory of Urban Land Resources Monitoring and Simulation, Ministry of Natural Resources, Shenzhen 518034, China; 2 Ministry of Education Key Laboratory for Earth System Modeling, Department of Earth System Science, Tsinghua University, Beijing 100084, China; luoxy17@mails.tsinghua.edu.cn (X.L.); xiaoyixiong@mail.tsinghua.edu.cn (Y.X.); yuqibai@tsinghua.edu.cn (Y.B.); 3 Tsinghua Urban Institute, Beijing 100084, China; 4 Shenzhen Research Center of Digital City Engineering, Shenzhen Municipal Bureau of Planning and Natural Resource Management, Shenzhen 518034, China; s_s_q@126.com; 5 School of Resource and Environment Science, Wuhan University, Wuhan 430079, China; 2014102050019@whu.edu.cn

**Keywords:** indicator system, health cities, evaluation, spatial distance, social media data

## Abstract

Various indicator systems have been developed to monitor and assess healthy cities. However, few of them contain spatially explicit indicators. In this study, we assessed four health determinants in Shenzhen, China, using both indicators commonly included in healthy city indicator systems and spatially explicit indicators. The spatially explicit indicators were developed using detailed building information or social media data. Our results showed that the evaluation results of districts and sub-districts in Shenzhen based on spatially explicit indicators could be positively, negatively, or not associated with the evaluation results based on conventional indicators. The discrepancy may be caused by the different information contained in the two types of indicators. The spatially explicit indicators measure the quantity of the determinants and the spatial accessibility of these determinants, while the conventional indicators only measure the quantity. Our results also showed that social media data have great potential to represent the high-resolution population distribution required to estimate spatially explicit indicators. Based on our findings, we recommend that spatially explicit indicators should be included in healthy city indicator systems to allow for a more comprehensive assessment of healthy cities.

## 1. Introduction

More than half of the global population is living in urban areas. While cities have brought convenience and opportunities to urban residents, cities also subject their residents to many health challenges. Non-communicable diseases are rising in urban areas because urban populations are exposed to an inactive lifestyle, an imbalanced diet, and urban environmental pollution [1,2,3]. Globalization has increased the connections among cities and facilitated the rapid spread of emerging infectious diseases among cities [4,5,6]. Cities worldwide are searching for solutions to these challenges. Since 1986, the Healthy Cities approach, which emphasizes the systematic management of the social and environmental determinants of health, has been proposed by the World Health Organization (WHO) as a systemic approach to deal with urban health challenges [7]. The approach will help a city to evolve into a Healthy City, which is defined as “one that is continually creating and improving those physical and social environments and expanding those community resources which enable people to mutually support each other in performing all the functions of life and developing to their maximum potential” [8]. The approach has gained popularity quickly all over the world. National and municipal governments are working with communities, non-governmental agencies, and private entities to launch many Healthy City projects. According to the WHO European Office, more than 1000 cities are now members of the European Healthy City Network [9]. Cities in Asia, South America, Africa are also actively building Healthy Cities and healthy communities [10,11,12]. The development of the Healthy City movement is uneven among different regions. European cities are leading the efforts to align the Healthy City movement with global efforts to achieve the sustainable development goals (SDGs) [13]. Cities in Europe have significantly extended the Healthy Cities approach beyond its original health mandate to address a broader development issue [9]. At the same time, other regions are holding to a more fundamental Healthy Cities approach. The Healthy City movement focuses on health equity, social justice, intersectoral collaboration, community participation, and public health in regions like Latin America and Asia [10,11,12].

The Healthy Cities approach involves a sequence of works, including a thorough analysis of the major health challenges and causes, the preparation of an action plan, the implementation of the plans, and the evaluation of progress. Monitoring and evaluation are indispensable parts of the Healthy Cities approach. Regular monitoring and evaluation allow cities to gauge the effectiveness of intervention measures and identify gaps when implementing the Healthy Cities approach [14,15]. The core of a monitoring and evaluation program is the indicator system, which contains indicators that measure the status of physical and social determinants of health and health outcomes in a city. Various Healthy City indicator systems have been developed over the past four decades. A review study counted a total of 145 urban health indicator systems and 8006 indicators [16]. The Healthy city indicators developed by the WHO European Office [17], the County Health Ranking System developed by the University of Wisconsin [18], and the China National Healthy City Indicators developed by the National Health Commission of China [19] are a few well-known examples. The numbers of indicators in these indicator systems varied, but their domains were mostly the same. These domains included socio-economic factors, physical environment factors, health behavior, health services, health outcomes, and organizational structures. Indicators that cover those domains form the backbone of indicator systems.

While the construction and application of Healthy City indicator systems have helped many cities to move towards being Healthy Cities, the choice of indicators for a city remains a challenging task. Indicators are expected to cover both the social determinants and physical attributes of a city. They are also expected to have many desirable features, such as being succinct, evidence-based, measurable, quantifiable, and accompanied by detail about their validity [20]. One issue that does not attract much attention is the lack of spatially explicit indicators in many Healthy City indicator systems. The physical and social determinants of human health in urban areas exist in space and time [21,22]. Nevertheless, indicators measuring these determinants were usually derived by summarizing the quantitative information at specific administrative levels, e.g., the number of hospital beds averaged by the population in a city or a district. This type of summarization works well if the determinant of interest distributes evenly in the study area, or everyone has equal access to the resource. In reality, both determinants and urban populations are known to concentrate in space [23,24,25], e.g., along traffic lines or a topographic feature such as a river. Therefore, the summarization process conceals the spatial relation between health determinants and the affected population. The failure to account for the spatial relation between health determinants and the urban population may lead to biased evaluation results. For example, a city with a vast park far from any population centers will get a higher mark than a city with many small parks dotted among its denser population if the area of parks per capita is the indicator used for evaluation.

Urban planners have long emphasized the importance of including spatially explicit indicators in urban planning and management [26,27,28,29]. The inclusion of spatial information explicitly when measuring the determinants of population health is also not a new idea. Etches et al. (2006) suggested “scalability” as a principle to select indicators to measure the multifactorial determinants of population health. The principle requires that data used for calculating indicators should correspond to the geographic level of the intervention, and the indicator can be produced for population subgroups [30]. Here, the population subgroups can be formed due to ethnicity, age, sex, and spatial locations. Pineo et al. (2018) listed “spatial” in the ten principles used to guide the development of the Building Research Establishment’s international Healthy Cities Index, which requires that “the indicators allow for data to be mapped spatially to inform urban planning policy and decision-making”. They also included eight spatially explicit indicators in the 58 indicators of their indicator system [20].

Despite the efforts to incorporate spatial information explicitly into Healthy City indicators, the rationale to have these spatially explicit indicators has not been thoroughly examined. Whether the extra effort needed to incorporate spatial information into Healthy City indicators is worthwhile is still an open question. This study attempts to address this question by comparing the difference between the evaluation results generated using conventional indicators and indicators derived by the spatially explicit approach. In the rest of this paper, we present the method for developing spatially explicit indicators and the results of comparisons through a case study conducted in Shenzhen, China. We also discuss the limitations of our study and the potential directions for future studies.

## 2. Datasets and Methodologies

### 2.1. Study Site

We chose Shenzhen, a city in south China, to conduct our case study. Shenzhen has gone through a rapid urbanization process over the past four decades. It started as a fishing village in 1979 and has grown into the modern industrial center of China. Shenzhen now consists of 11 districts and 74 sub-districts, with an administrative area of 1997.47 km^2^. Shenzhen’s built area has reached 927.96 km^2^, while the size of the resident population reached 13.03 million by 2018 [31]. The top health challenges faced by Shenzhen’s citizens are non-communicable diseases, such as malignant tumors, heart diseases, and cerebrovascular disease (CVD), which were the major causes of deaths in Shenzhen in 2019 [32].

### 2.2. Data Collection

Based on data availability, we selected four categories of health determinants listed in the urban health indicator framework developed by Pineo et al. [20] for evaluation in this study, including green infrastructure, transportation, utilities and services, and leisure and recreation.

We obtained the resident population (people who had lived in Shenzhen six months or longer in the 2015 national census) and locations of residential buildings, green spaces (natural and human-made green spaces), public transit stops and stations, and community health centers from the Shenzhen Research Center of Digital City Engineering, Shenzhen Municipal Bureau of Planning and Natural Resource Management. The population data at the sub-district level was created by downscaling census data at the district level and ancillary information. All data were provided to us in the format of point and polygon ArcGIS shapefiles. We excluded the Shenshan Special Collaboration Zone from the analysis as it is a district in another city but administered by the Shenzhen government.

In addition to the official data, we also obtained point of interest (POI) data for the entirety of Shenzhen in 2018 from the largest online map provider in China—Gaode—and extracted the sports facility locations. In this study, sports facilities refer to all stadiums, sports grounds, fitness centers, swimming pools, and other indoor and outdoor places people use for physical exercise. We acquired the Tencent user density (TUD) data at 10 pm on 29 August 2019 from Easygo. The data is a digital map (ground resolution = 24 m) showing the number of social network users on Tencent. Tencent is the largest online social media platform in China, which had more than 1.15 billion users by 2019—about 82% of China’s total population (1.4 billion). Due to its massive user base, the TUD data can provide a real-time representation of population distribution. We assumed that data obtained at night captured people who lived in the sites.

### 2.3. Data Analysis

We adopted an indicator from existing health indicator systems for each health determinant category and calculated its value accordingly (Table 1). We then estimated the indicator using a spatially explicit approach. Shenzhen promotes a 15 min living radius program to provide facilities to satisfy residents’ daily life needs within a 15 min walking distance to their residences. Studies found that people could cover 1.0–1.4 km by walking in 15 min, varying by age and environment [33,34]. Therefore, we chose the lower-end value of 1.0 km to represent this distance to consider every age group. We estimated the percentage of the population living within this distance to a specific type of facility. Because the high-resolution map of population distribution was not publicly available in Shenzhen, we adopted two alternative approaches to deal with this data gap.

First, we followed existing studies by using residential buildings as a substitute for the population distribution. For example, the WHO European Office recommends using the percentage of the population that is less than 300 m from a green space with a minimum size of 1 ha as an indicator of green spaces [35]. Pineo et al. (2018) replaced the population with dwellings [20]. We slightly modified the method to reflect the local situation in Shenzhen. Instead of using dwellings, we used residential buildings’ floor areas because many residential buildings in Shenzhen are high-rise (≥10 floors) or super high-rise buildings (≥30 floors). Using a building as the basic unit in the calculation will add biases to the result. Besides using the floor areas of residential buildings, we used the TUD data as a proxy of the population (hereafter, the WeChat population). The TUD data are directly associated with the population density in space. Therefore, we assumed that the percentage of counts inside a 1 km radius of a facility to all counts in a sub-district or district could approximate the percentage of the population living within a 1 km radius of that facility for the total population.

All indicators were estimated by first calculating the spatial distances among facilities, people, and residential buildings with the ArcGIS’s near and overlay functions (Version 10.6, ESRI, Redlands, CA, USA). To reveal the effect of aggregating the indicator to different geographic levels, we summarized the indicator values at the sub-district level to the district level. After calculation, we ranked each district or sub-district by the indicator values. We compared the rankings rather than the raw values of the indicators because the raw values were not directly comparable as they were in different dimensions and units; e.g., the number of doctors per 100,000 people versus the percentage of floor areas of residential buildings in 1000 m of community health centers. To resolve this issue, we used rankings of sub-districts or districts in comparisons. The method has practical value and a statistical root. In practice, policymakers are more interested in rankings than raw values because the former provides them a sense of performance. The use of rankings in Healthy City assessments has been practiced in many cities [36,37,38]. Statistically, it is common to use ranking-based tests when the data violate the underlying statistical assumption. The differences in rankings were examined using Spearman’s rank-order correlation, which measures the association between rankings. A correlation value of +1 or −1 indicates a perfect positive or negative association of rankings, while a value of zero indicates no association at all between rankings.

## 3. Results

### 3.1. Indicators of Green Infrastructure

Overall, Shenzhen had 921.2 km^2^ of green spaces by 2016. The per capita value was 77.36 m^2^/person. In total, 48.6% of the floor areas of residential buildings were located in 300 m of green space (≥1.0 ha), and 43.84% of the WeChat population was in that range.

At the district level, the values of indicators varied in a wide range (Table 2). The Dapeng district, the most remote district in Shenzhen, had the highest per capita value. The rankings based on the conventional indicator were positively correlated with the rankings based on the indicator of building floor areas (ρ = 0.85, *p*-value = 0.003505) and the rankings based on the indicator of WeChat populations (ρ = 0.89, *p*-value = 0.00138). The positive correlation between two new indicators was strong (ρ = 0.94, *p*-value < 2.2 × 10^−16^).

**Figure 1:**
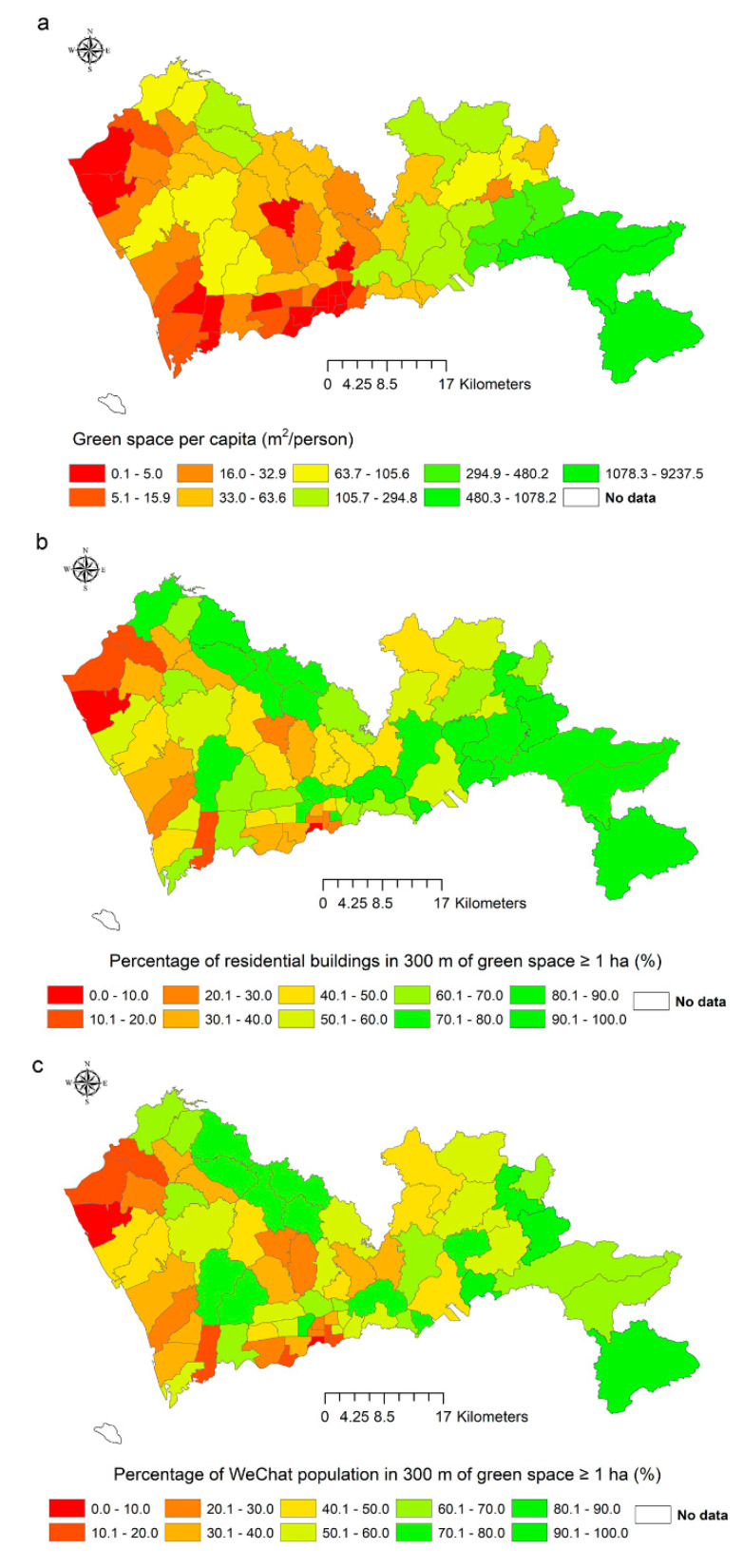
The indicator values of green spaces in 74 sub-districts. (**a**) The area of green space per capita; (**b**) the percentage of residential buildings <300 m to a green space with a minimum size of 1 ha; (**c**) The percentage of WeChat population <300 m to a green space with a minimum size of 1 ha.

The values of the three indicators at the sub-district level differed substantially (Figure 1). The rankings of the sub-districts based on the conventional indicator were less correlated to rankings based on the spatially explicit indicators (ρ = 0.68, *p*-value < 2.2 × 10^−16^; ρ = 0.72, *p*-value < 2.2 × 10^−16^). However, the two spatially explicit indicators still had strong correlations (ρ = 0.97, *p*-value < 2.2 × 10^−16^).

### 3.2. Indicators of Transportation

Shenzhen had 8299 bus stops and subway stations by 2016, i.e., around 6.96 stops or stations per 10,000 people. Nearly 99.47% of floor areas of residential buildings were located within a 1000 m distance of a stop or station, and 99.2% of the WeChat population lived in that range.

At the district level, the rankings based on the conventional indicator correlated negatively with the rankings based on the spatially explicit indicators (Table 3). The correlation between the rankings based on the conventional indicator and the rankings based on the indicator of residential buildings was significant (ρ = −0.83, *p*-value = 0.005557). In contrast, the correlation between the conventional indicator and the rankings based on the indicator of the WeChat population was not significant at the 95% confidence level (ρ = −0.75, *p*-value = 0.01841). The two new indicators again showed a strong positive correlation (ρ = 0.92, *p*-value = 0.0004667).

**Figure 2:**
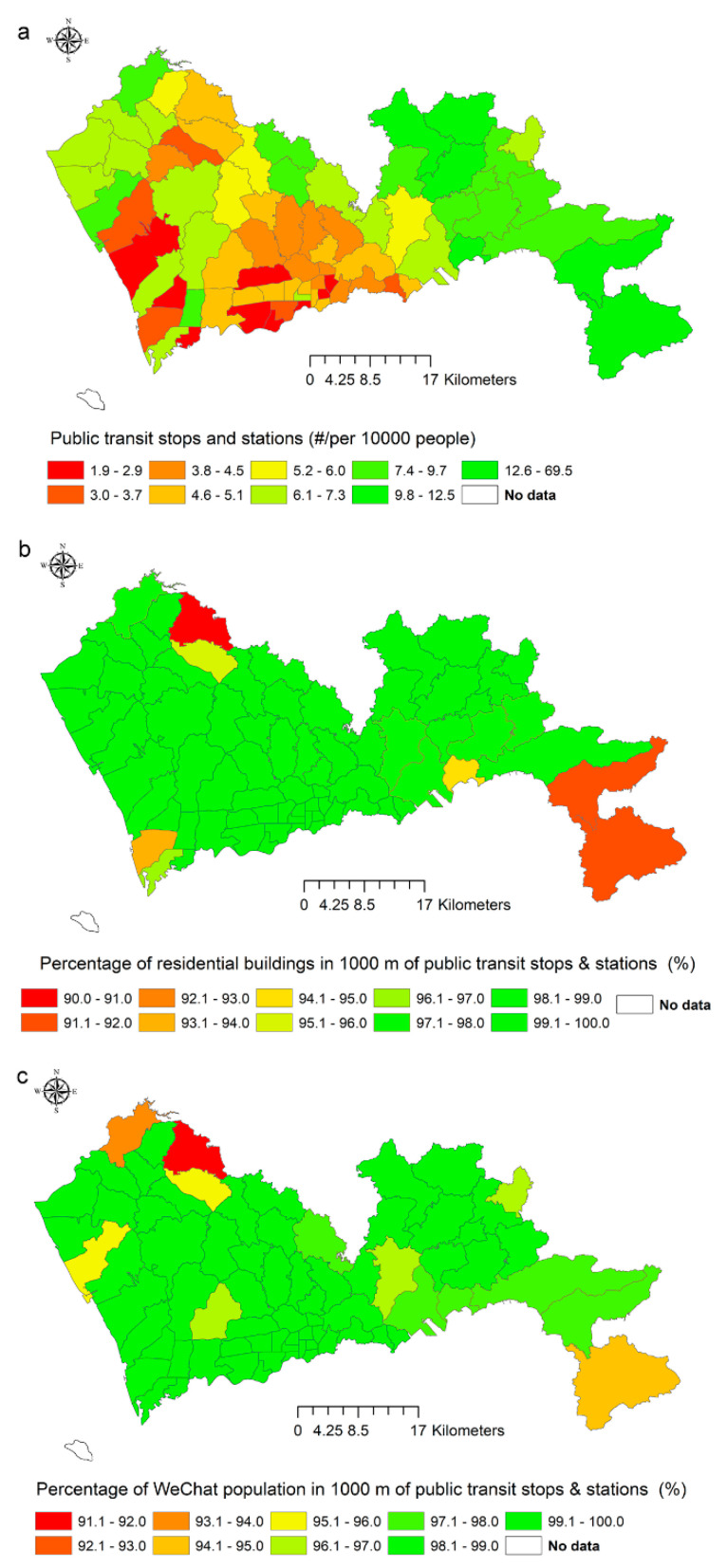
Values of the indicator of transportation in 74 sub-districts. (**a**) The number of public transit stops and stations per 10,000 people; (**b**) the percentage of residential buildings ≤1000 m to public transit stops and stations; (**c**) the percentage of WeChat population ≤1000 m to public transit stops and stations.

At the sub-district level, the main differences were again between the conventional indicator and the two new indicators (Figure 2). The rankings based on the conventional indicator correlated negatively with the rankings based on the indicator of residential buildings (ρ = −0.43, *p*-value = 0.000219) and the rankings based on the indicator the WeChat population (ρ = −0.47, *p*-value = 5.4 × 10^−5^). The correlation between the two spatially explicit indicators was still positive and significant (ρ = 0.75, *p*-value = 1.2 × 10^−10^).

### 3.3. Indicators of Utilities and Services

In Shenzhen, 589 community health centers hosted 3280 doctors by 2016, which amounted to 2.75 doctors in community health centers per 10,000 permanent residents. City-wide, 90.85% of residential buildings’ floor areas were located within a 1000 m distance to a community health center, and 88.07% of the WeChat population lived in that distance.

At the district level (Table 4), the rankings based on the conventional indicator were not significantly correlated with the rankings based on the indicator of residential buildings (ρ = 0.02, *p*-value = 0.9728) as well as the rankings based on the indicator of WeChat populations (ρ = −0.04, *p*-value = 0.9186). The two spatially explicit indicators were significantly associated (ρ = 0.92, *p*-value = 0.0004667).

**Figure 3:**
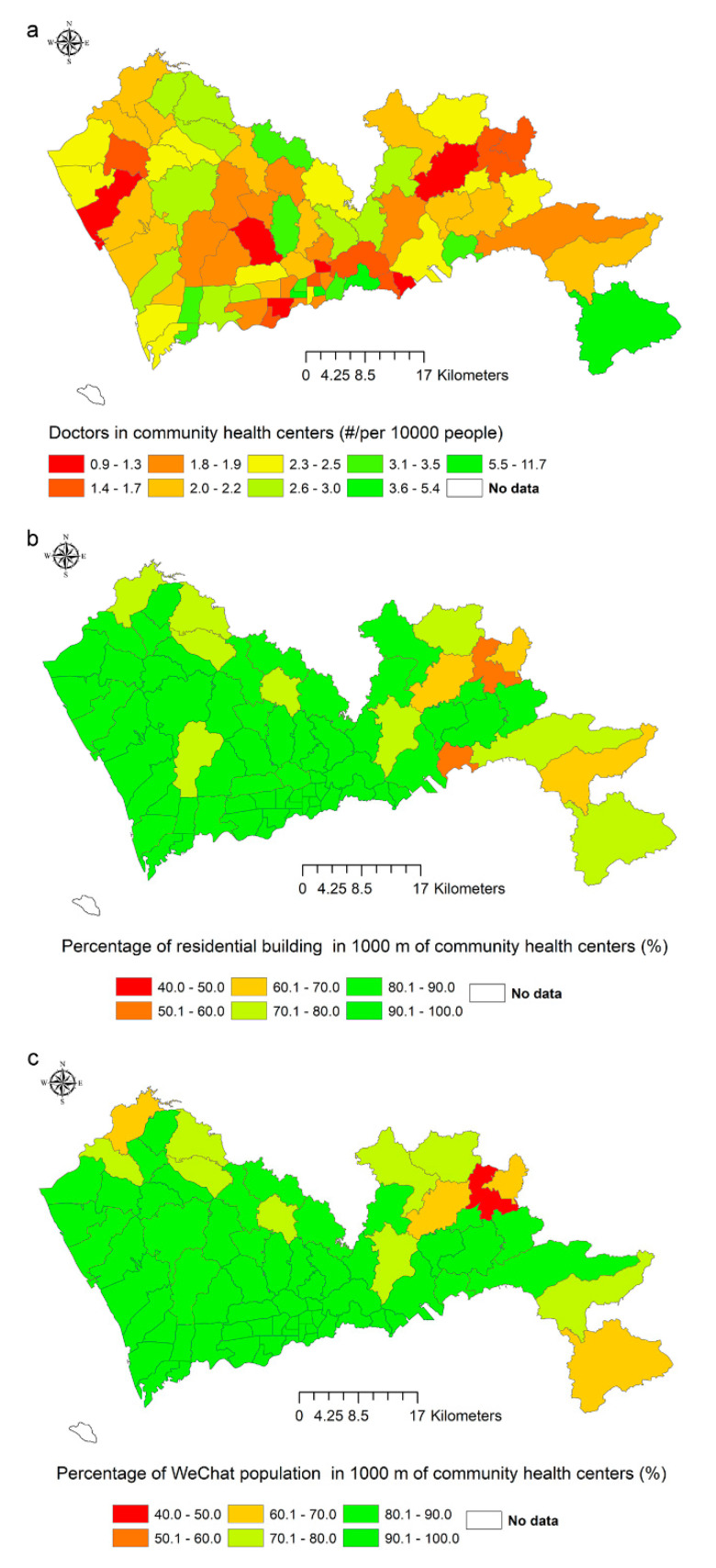
Values of the indicator of health services in 74 sub-districts. (**a**) The number of doctors in community health centers per 10,000 people; (**b**) the percentage of residential buildings ≤1000 m to community health centers; (**c**) the percentage of WeChat population ≤1000 m to community health centers.

The conventional indicators revealed a different spatial pattern at the sub-district level compared to the patterns revealed by the two new indicators (Figure 3). The conventional indicator values showed that sub-districts that were doing well were interspersed with the low performers across the city, while the values of spatially explicit indicators suggested that low performers were concentrated in the southeast part of the city. The correlations were not significant between the rankings based on the conventional indicator and the rankings based on the spatially explicit indicators (ρ = −0.06, *p*-value = 0.605; ρ = −0.02, *p*-value = 0.895). The correlation between the two new indicators was still significantly strong (ρ = 0.92, *p*-value = 3.8 × 10^−15^).

### 3.4. Indicators of Leisure and Recreation

Shenzhen had around 8300 sports facilities in 2018, with roughly 6.97 facilities per 10,000 people. About 98.23% of floor areas of residential buildings and 97.8% of the WeChat population were located within a 1000 m distance to at least one sports facility.

At the district level, the rankings based on the conventional indicator correlated significantly with the rankings based on the two spatially explicit indicators (Table 5), with the values of correlation coefficients were ρ = 0.66 and *p*-value = 0.04403, and ρ = 0.67 and *p*-value = 0.03938 for the indicator based on building floor areas and the indicator based on WeChat population, respectively. However, the correlation between the two spatially explicit indicators was significantly strong (ρ = 0.99, *p*-value < 2.2 × 10^−16^).

**Figure 4:**
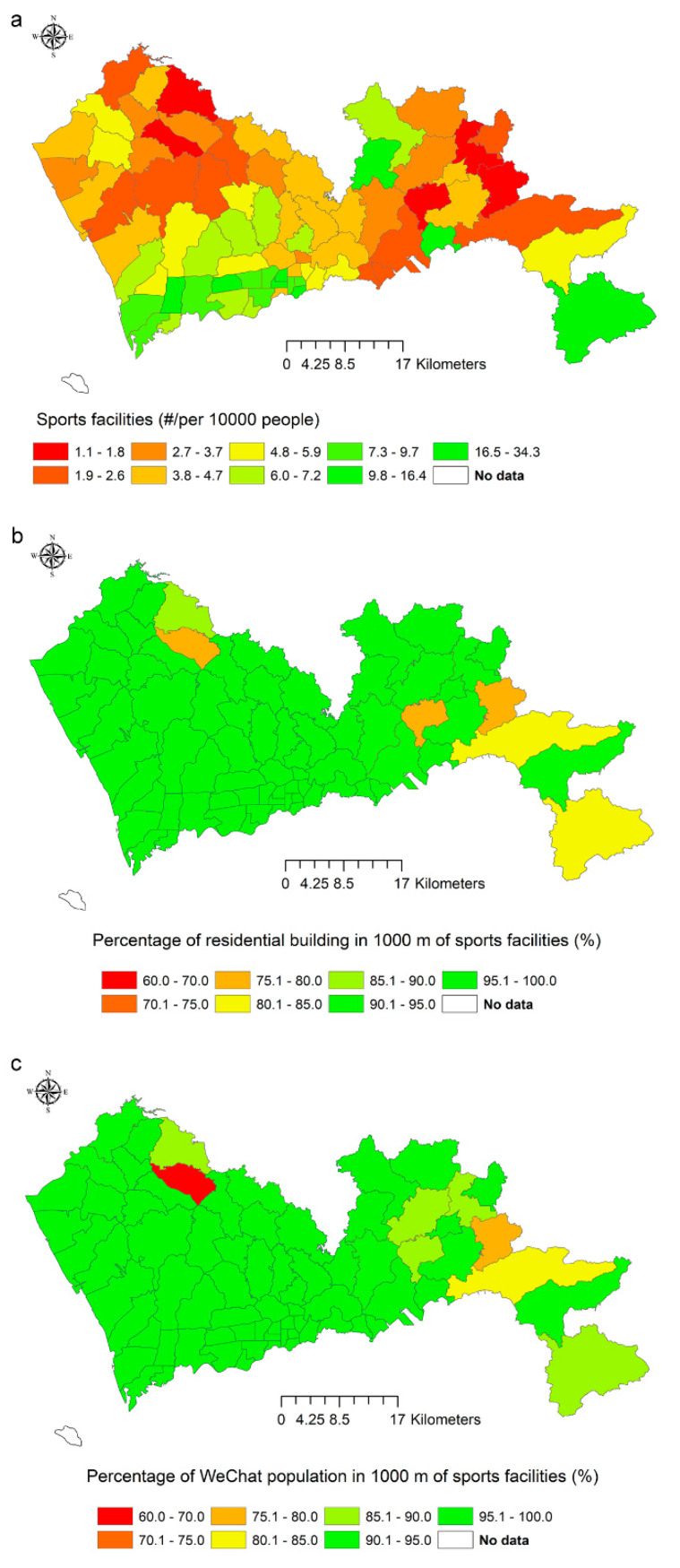
Values of the indicator of sports facilities in 74 sub-districts. (**a**) The number of sports facilities per 10,000 people; (**b**) the percentage of residential buildings ≤1000 m to sports facilities; (**c**) the percentage of WeChat population ≤1000 m to sports facilities.

At the sub-district level, the conventional indicator values differed from two spatially explicit indicators in most sub-districts (Figure 4). The rankings based on the conventional indicator were positively correlated with the rankings based on the two spatially explicit indicators (ρ = 0.52, *p*-value = 7.4 × 10^−6^; ρ = 0.58, *p*-value = 6.5 × 10^−7^). The correlation between the two new indicators was still strong (ρ = 0.91, *p*-value < 7.8 × 10^−15^).

### 3.5. Indicators of Utilities and Services

**Figure 5:**
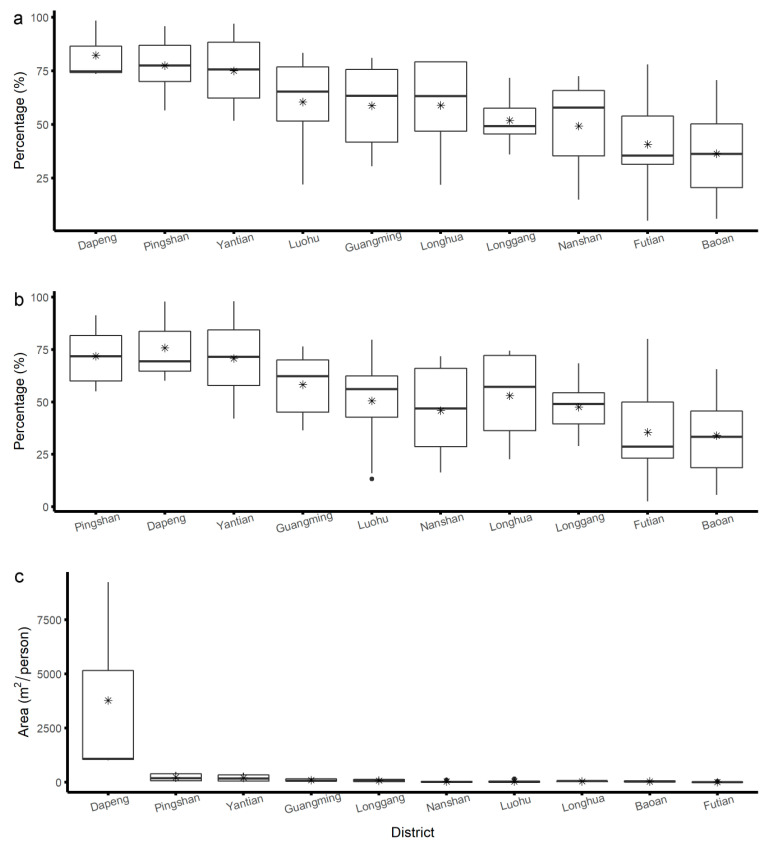
The box plots show the values of green infrastructure indicators at the sub-district level grouped by each district. The districts were ordered according to the rankings at the district level. The lower end of the box represents the first quartile, and the upper end represents the third quartile. The ends of the whiskers represent 1.5 times the interquartile range. The stars are mean values. (**a**) The percentage of residential buildings <300 m to a green space with a minimum size of 1 ha; (**b**) the percentage of WeChat population <300 m to a green space with a minimum size of 1 ha; (**c**) the area of green space per capita.

By grouping the indicator values of each sub-district by districts, the variation of the indicator value at the sub-district level in each district was revealed. Figure 5 shows an example of green infrastructure (figures for other indicators can be found in the Supplementary Materials). The figure showed that the two spatially explicit indicators were less affected by extreme values.

## 4. Discussion

### 4.1. Comparing Conventional Indicators and Spatially Explicit Indicators

Including the spatial information explicitly into the Healthy City indicators can affect the evaluation results significantly. Our study identified three kinds of situations: (1) the evaluation results based on the two types of indicators agreed with each other; (2) the evaluation results based on the conventional indicator had little relevance with the evaluation results based on the spatially explicit indicators; and (3) the evaluation results based on the two types of indicators were opposite to each other.

The indicators that we used to measure urban green infrastructure and recreation correspond to the first situation. The correlations between the rankings reached using the conventional indicator and the rankings reached using the spatially explicit indicators were positive and significant, indicating that the ranking results mostly agreed. The conventional indicator—i.e., green space per capita—was derived using the total area of green spaces divided by the population in an individual administrative unit. This indicator conveys quantitative information on the availability of green spaces in that unit. The two spatially explicit indicators—i.e., the percentage of residential building floor areas or the WeChat population less than 300 m to a green space with a minimum size of 1 ha—were modified from the green space indicator proposed by the WHO European Office. The WHO European Office suggests that the indicator should measure the accessibility of green spaces defined by spatial distances [35]. In densely populated districts or sub-districts, the shortage of green spaces was reflected in the availability and accessibility of green spaces. In sparsely populated and well-planted districts or sub-districts, the availability of many green spaces could increase the accessibility of green spaces to residents. Nevertheless, the values of correlation coefficients were not near +1, especially at the sub-district level, which indicated a divergence between the availability and accessibility of green spaces. The indicators measuring sports facilities showed a similar pattern, but the correlations between the rankings based on the conventional indicator and the rankings between the spatially explicit indicators were weaker than those of the former. The difference may be explained by the fact that green spaces were more ubiquitous around residences than sports facilities, as the former are usually included in housing developments. Voorde (2017) suggested that spatially explicit urban green indicators are more meaningful for deriving statistics at the city level or the neighborhood level [39]. Our study provides methods for deriving this kind of indicator.

The indicators that measure health service provision by community health centers correspond to the second type of situation. The near-zero values of the correlation coefficients indicated that evaluation results based on the two types of indicators were not associated. The number of doctors averaged by population is commonly used to indicate the availability of health services [17,18,19]. Nevertheless, this indicator does not reflect the unique role of community health centers well. Those centers are assigned gatekeepers’ role in the hierarchical health care system that China is working hard to establish. The government expects residents, especially those with chronic diseases, to use community health centers as the first stop when seeking health services [40,41]. To fulfill that purpose, these centers have to be located in places where they can be easily accessed, preferably within walking distance. Indicators that measure the accessibility of community health centers are, therefore, essential to evaluate this function. The lack of association between the two types of indicators means that both indicators are needed in the evaluation as they measure different aspects of the provision of health services from community health centers.

The third situation includes indicators that measure transportation. The evaluation results based on conventional indicators were negatively associated with the evaluation results based on spatially explicit indicators. The opposite result indicated that more transit stops and stations per unit population did not automatically translate into better transportation accessibility. For example, Dapeng District had the highest number of public transit stops and stations per 10,000 people but had the lowest percentage of residential building floor areas and WeChat population in a 1000 m radius of public transit stops and stations. The sparsely distributed population and the mountainous topography in the district made it difficult for a stop or station to serve many people. In contrast, Futian District had the lowest number of public transit stops and stations per 10,000 people, but the percentage of residential building floor areas in the 1000 m radius of public transit stops and stations was 100%. As the city center of Shenzhen, the district has the highest population density, which means a transit stop or a station can serve many people. The opposite evaluation results highlighted the risk of obtained biased evaluation results if spatially implicit indicators are relied upon to evaluate Healthy Cities. Our findings echoed the findings from several public transportation studies that accessibility should be a primary concern when planning for public transportation [42,43].

By viewing the variation of indicator values at the sub-district level and the district level together, we found that the spatially explicit indicators were less affected by the extreme values when aggregation of the values at the sub-district level to the district level. The spatially explicit indicators are based on the relative spatial distances of facilities to residents in a specific area. They would not change with the unit size used in the assessment as long as the spatial relation between the facilities and the population stays the same, which is a desirable property of Healthy City indicators. This feature avoids the Modifiable Areal Unit Problem in Healthy City indicators [20], which is a statistical biasing effect whereby the same basic data yield different results when aggregated in different ways. Furthermore, spatially explicit indicators alleviate the situation in which the poor performance of many units could be averaged out by a few good performers when scaling the indicators up to a higher geographic level. Conventional indicators are more prone to these problems as they are often averaged over a geographic area.

Our study found that the evaluation results based on the two spatially explicit indicators were strongly associated with each other. This finding suggests that social media data, especially data associated with residents’ daily life, such as data from WeChat, can serve as a proxy of high-resolution population distribution. High-resolution population distribution data are not publicly available in most countries. In China, the national census data in China only include the resident population, the registered population, and non-registered population. For a megacity such as Shenzhen, where the floating population size is almost the same as the resident population, the official population data do not reflect the real population distribution. The highest resolution of the official population data available to the public is at the sub-district level. The data are not very useful when estimating spatially explicit indicators that require high-resolution spatial information.

Detailed information on residential buildings’ locations and floor areas can paint a better picture of the population distribution in space. Nevertheless, this kind of information is difficult to obtain in many cities due to poor records or restrictions. Furthermore, residential buildings’ floor areas do not correlate with the number of residents perfectly, as people can cram into small spaces or leave rooms unoccupied. Our study showed that the WeChat population data could provide a potential solution to this data problem. Although we only used the WeChat population data for one night in our study due to budget difficulties, the results were already highly comparable to results obtained using the detailed building data. We believed that the results could be further improved using more extended time series accounting for variations in weekends and seasons. Since the datasets are commercially available, they hold great potential for deriving Healthy City indicators that require high-resolution population distribution data. While WeChat data are most suitable for China, similar social media data such as Twitter may be explored in other countries.

### 4.2. Implications for Healthy City Practices

The findings from our study have implications on the task of selecting Healthy City indicators in general. To date, most healthy city indicators only provide the quantity information of health determinants, such as the number of hospital beds or areas of green spaces. The use of these indicators in evaluations can stimulate cities to increase the supply of facilities or services to achieve better rankings [44]. Nevertheless, as shown in our study, more facilities may not always translate into higher accessibility. This situation is especially a concern for people whose mobilities are restricted for physical or socio-economic reasons, such as the elderly, disabled people, or people living in poverty [45]. The rush to add more facilities such as parks and sports grounds has often resulted in locating these facilities in well-to-do communities because they have financial resources and political influences, further aggravating the health inequality issue that the Healthy Cities approach aims to resolve [46]. Therefore, spatially explicit indicators are necessary for the evaluation of Healthy Cities. To develop a coherent and reasonably comprehensive set of Healthy City indicators, researchers or practitioners should strive to cover the main domains of Healthy City indicators with spatially explicit indicators whenever they can.

Our findings also had implications for urban planning and management in Shenzhen. Among the four indicators evaluated in this study, Shenzhen is doing well in providing public transport and recreational facilities. Except for a remote district, more than 98% of the population had at least one facility within a 1000 m distance. However, the performance of Shenzhen in the other two indicators can be improved. If the spatially explicit indicator proposed by the WHO European Office is used, the distribution of urban green spaces in Shenzhen was not optimal. Some densely populated districts would need creative ways to provide a sufficient amount of green spaces for various recreational activities. Furthermore, when building community health centers in Shenzhen in the future, attention should be paid to increasing the number of centers and doctors and locating them strategically to increase accessibility. Finally, at the subdistrict level, the accessibility of transportation, health, and sports facilities declined from south to north and from east to west. More efforts should be made to address inequality among sub-districts.

### 4.3. Limitations of the Current Study

While our study clearly showed the necessity of including spatially explicit indicators in Healthy City indicators, some limitations of this study should be further addressed in future research. First, we used the spatial distance to estimate the values of spatially explicit indicators due to its simplicity. The simplification is adequate to reveal the difference between the two types of indicators: our study’s primary goal; however, using the travel distance in estimation will generate more realistic results [47]. Second, adding spatially explicit indicators into Healthy City indicators should only be viewed as an intermediate step. A facility’s accessibility is affected by its distance to users and the myriad social and economic factors [48,49]. A better indicator of a facility’s impact on urban residents’ health is the population that uses it daily. Social media data and other types of big data again have great potential for estimating the indicator of actual usage as they can provide information on how urban residents use and respond to the infrastructures and built environment in cities [50]. However, users should keep in mind the limitations of social media data. For example, while WeChat data cover a high proportion of the Chinese population, certain population groups such as young children are not covered. Social media data need to be used with caution.

## 5. Conclusions

Indicators are indispensable for Healthy Cities to gauge progress and identify gaps in making informed policies and taking targeted actions. The commonly used indicators mainly measure the quantity dimension of health determinants. These indicators do not reflect the spatial relation between residents and these determinants well. In this study, we showed that the inclusion of spatially explicit indicators could bring new insights that conventional indicators fail to deliver by comparing the evaluation results based on spatially explicit and implicit indicators. We also showed that social media data could be used as a proxy of high-resolution population distribution data when calculating spatially explicit indicators. Based on our results, we recommend that spatially explicit indicators be included in future evaluations of healthy cities to understand the health determinants in those cities better.

## Figures and Tables

**Table 1:** Healthy City indicators and spatially explicit counterparts used in this study and the ways to calculate them.

Determinants	Conventional Indicators	Indicator Based on Building Floor Areas	Indicators Based on WeChat Populations
Green infrastructure	Green space per capita (m^2^/person)	Percentage of floor areas <300 m from green space with a minimum size of 1 ha (%)	Percentage of the WeChat population <300 m from green space with a minimum size of 1 ha (%)
Transportation	Number of transit stops and stations per 10,000 resident population (no./10,000 people)	Percentage of floor areas in 1000 m distance to a transit stop or station (%)	Percentage of the WeChat population in 1000 m distance to a transit stop or station (%)
Utilities and services	Number of doctors in community health lefts per 10,000 resident population (no./10,000 people)	Percentage of floor areas in 1000 m distance to a community health left (%)	Percentage of the WeChat population in 1000 m distance to a community health left (%)
Leisure and recreation	Sports facilities per 10,000 resident population (no./10,000 people)	Percentage of floor areas in 1000 m distance to a sports facility (%)	Percentage of the WeChat population in 1000 m distance to a sports facility (%)

**Table 2:** Ranking of districts by the indicators of green infrastructure.

District	Conventional Indicator (m^2^/person)	Ranking	Indicator Based on Building Floor Areas (%)	Ranking	Indicator Based on Wechat Populations (%)	Ranking
Baoan	35.26	9	33.48	10	32.13	10
Dapeng	1751.20	1	78.09	1	67.58	2
Futian	13.18	10	41.87	9	35.03	9
Guangming	118.09	4	55.46	5	55.79	4
Longgang	80.31	5	50.61	7	46.58	8
Longhua	38.78	8	53.05	6	46.85	7
Luohu	41.22	7	59.35	4	49.02	5
Nanshan	43.06	6	48.54	8	47.60	6
Pingshan	245.36	2	74.82	2	69.84	1
Yantian	220.84	3	70.62	3	62.25	3

**Table 3:** Rankings of districts by the indicator of transportation.

District	Conventional Indicator (#/per 10,000 People)	Ranking	Indicator Based on Building Floor Areas (%)	Ranking	Indicator Based on WeChat Populations (%)	Ranking
Baoan	6.94	5	99.66	5	98.87	7
Dapeng	16.11	1	95.20	10	97.40	10
Futian	4.32	10	100.0	1	99.97	1
Guangming	6.94	6	98.18	9	98.49	9
Longgang	8.44	3	99.25	6	99.18	5
Longhua	6.77	7	99.88	4	99.91	2
Luohu	5.12	9	99.90	3	99.86	3
Nanshan	6.73	8	99.94	2	99.20	4
Pingshan	11.77	2	98.94	7	98.94	6
Yantian	7.68	4	98.27	8	98.78	8

**Table 4:** Rankings of districts by the indicator of health services.

District	Conventional Indicator (Doctor/per 10,000 People)	Ranking	Indicator Based on Building Floor Areas (%)	Ranking	Indicator Based on WeChat Populations (%)	Ranking
Baoan	2.56	8	92.79	4	88.29	5
Dapeng	2.84	5	70.82	10	81.94	9
Futian	2.57	7	97.89	2	98.61	2
Guangmin	3.35	1	87.85	7	86.80	7
Longang	2.90	4	87.33	8	84.38	8
Longhua	2.24	10	90.85	5	87.91	6
Luohu	3.27	2	98.0	1	99.06	1
Nanshan	3.22	3	94.81	3	90.58	4
Pingshan	2.72	6	75.04	9	73.08	10
Yantian	2.52	9	89.02	6	93.46	3

**Table 5:** Rankings of districts by the indicator of leisure and recreation.

District	Conventional Indicator (#/per 10,000 People)	Ranking	Indicator Based on Building Floor Areas (%)	Ranking	Indicator Based on WeChat Populations (%)	Ranking
Baoan	5.03	7	99.47	4	98.31	5
Dapeng	6.46	5	85.22	10	87.95	10
Futian	10.02	2	100.0	1	100.0	1
Guangmin	4.23	9	93.67	8	93.97	8
Longang	6.93	4	97.85	7	97.3	7
Longhua	5.29	6	99.07	5	98.9	4
Luohu	7.86	3	99.93	2	99.89	2
Nanshan	11.76	1	99.61	3	99.31	3
Pingshan	3.68	10	91.73	9	92.14	9
Yantian	4.90	8	98.04	6	97.79	6

## Supplementary Materials

The following are available online at https://www.mdpi.com/1660-4601/17/20/7409/s1, Figure S1: Indicators of transportation, Figure S2: Indicators of health service, Figure S3: Indicators of recreation.
