# Supplementary material for: Comparing the Use of Spatially Explicit Indicators and Conventional Indicators in the Evaluation of Healthy Cities: A Case Study in Shenzhen, China
Source: Int J Environ Res Public Health. 2020 Oct 12;17(20):7409. doi: 10.3390/ijerph17207409 (PMC7601529; doi:10.3390/ijerph17207409)
Supplement: Supplementary file 1 [file ijerph-17-07409-s001.pdf]

**Supplementary Materials**

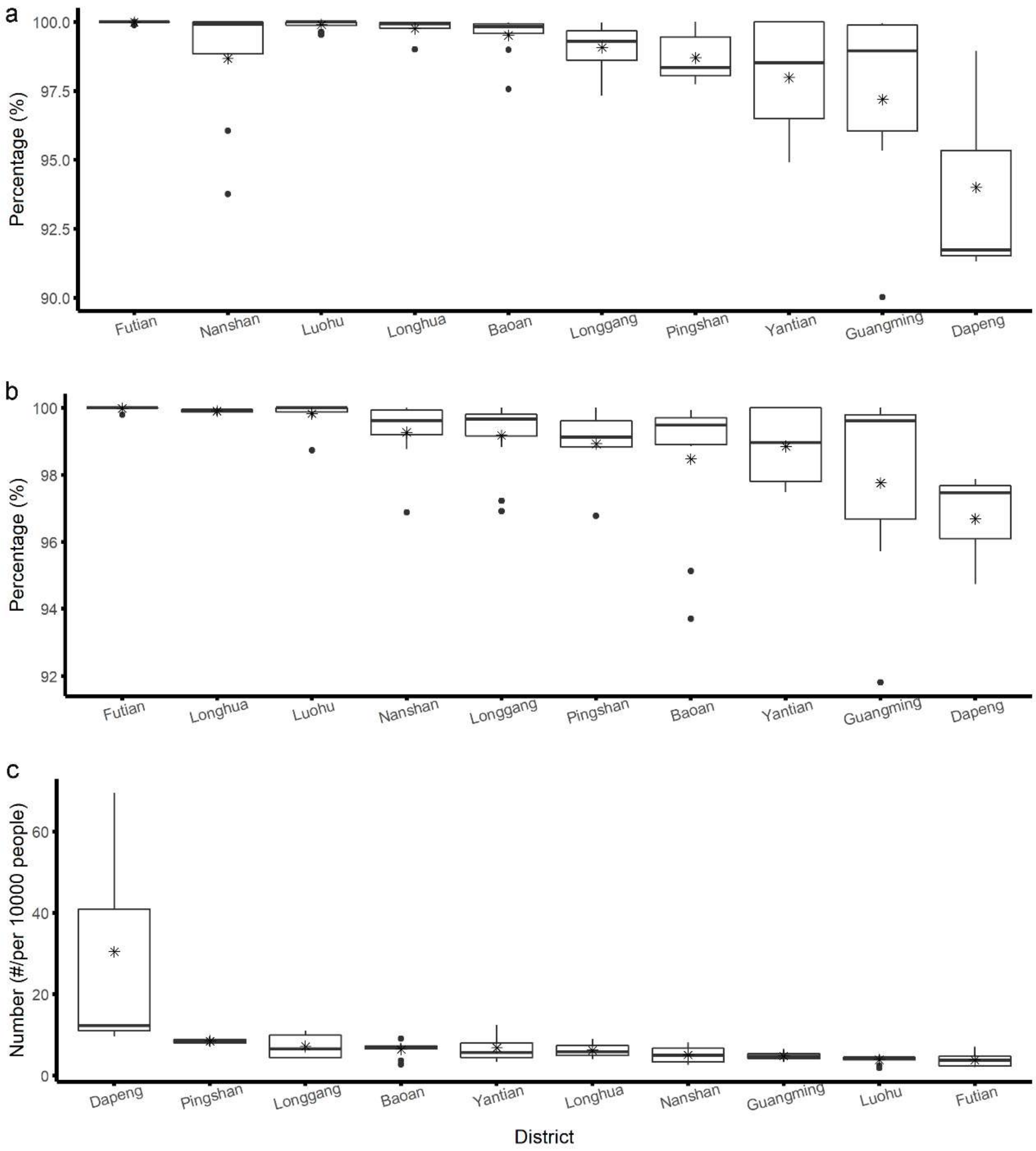


**Figure S1.** Values of indicators of transportation at the sub-district level grouped by each district. The districts were ordered according to the rankings at the district level. The stars are mean values. (**a**) The percentage of residential buildings ≤1000 m to public transit stops and stations; (**b**) The percentage of WeChat population ≤1000 m to public transit stops and stations (**c**) Number of public transit stops and stations per 10,000 people.

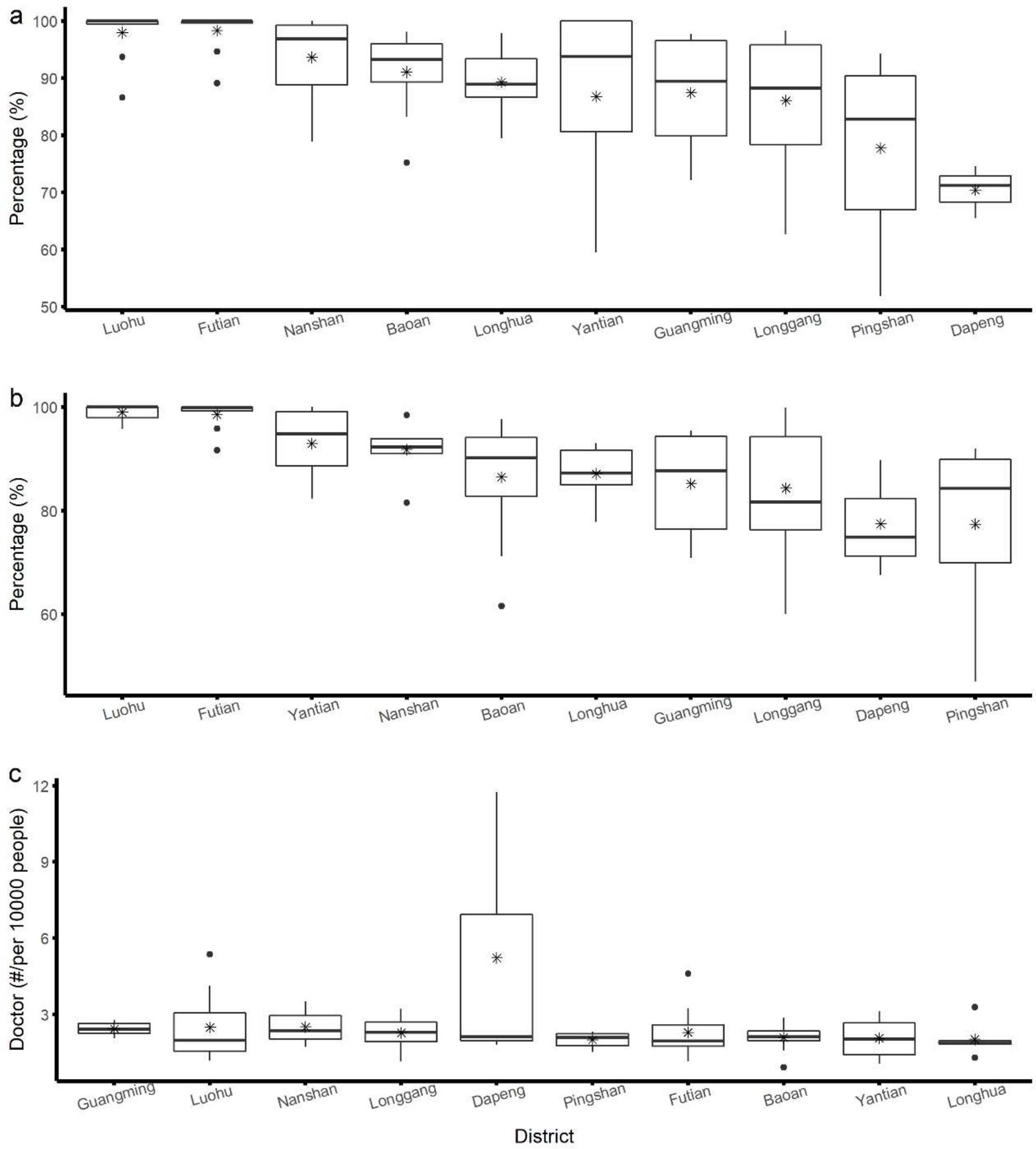


**Figure S2.** Values of indicators of health services at the sub-district level grouped by each district. The districts were ordered according to the rankings at the district level. The stars are mean values. (**a**) The percentage of residential buildings ≤1000 m to community health centers; (**b**) The percentage of WeChat population ≤1000 m to community health centers; (**c**) Number of doctors in community health centers per 10,000 people.

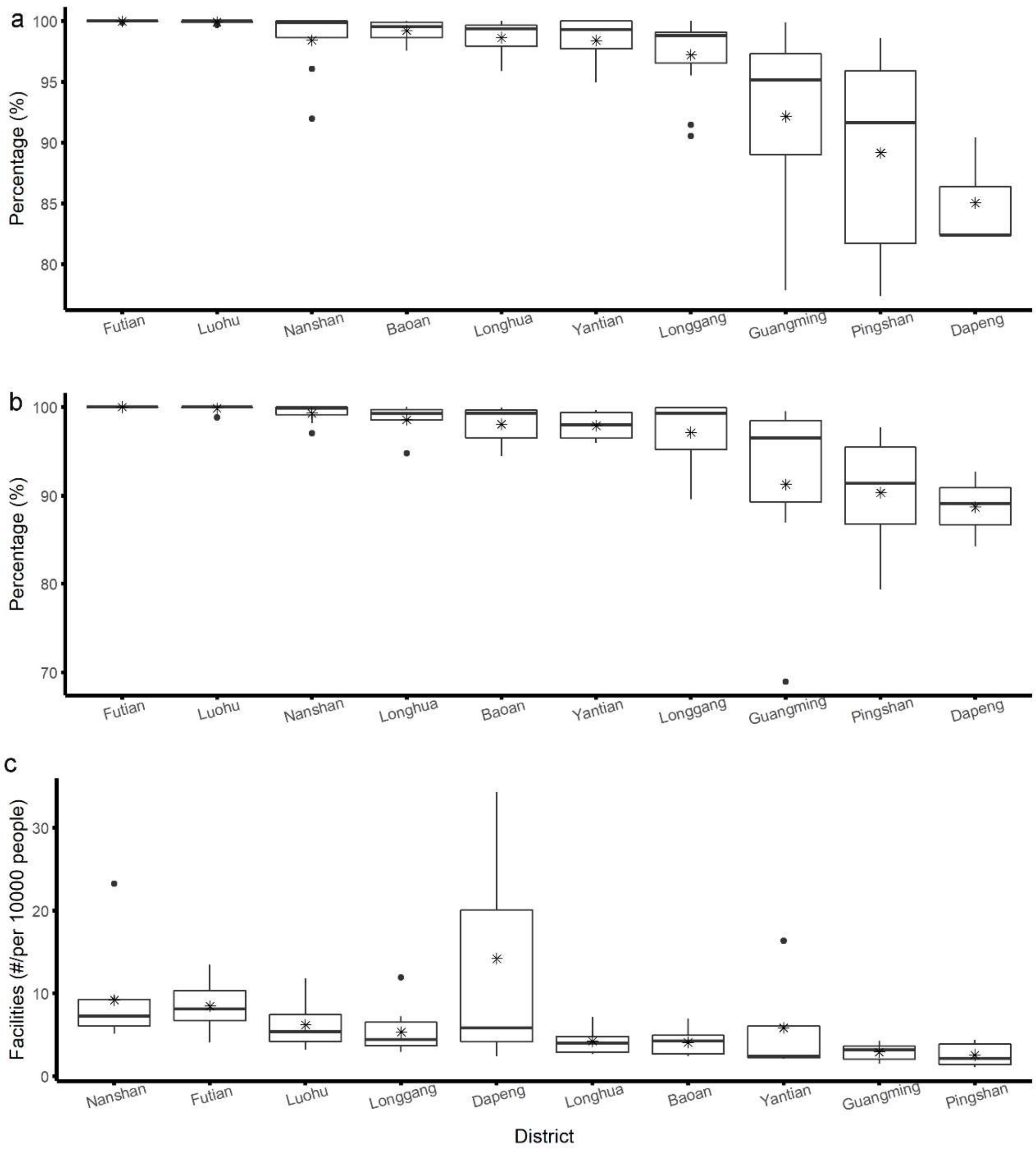


**Figure S3.** The values of indicators of sports facilities at the sub-district level grouped by each district. The districts were ordered according to the rankings at the district level. The stars are mean values. (**a**) The percentage of residential buildings ≤1000 m to sports facilities; (**b**) The percentage of WeChat population ≤1000 m to sports facilities; (**c**) Number of sports facilities per 10,000 people.
